# Green synthesis of multifunctional MgO@AgO/Ag_2_O nanocomposite for photocatalytic degradation of methylene blue and toluidine blue

**DOI:** 10.3389/fchem.2022.1083596

**Published:** 2022-12-15

**Authors:** Younes Zidane, Salah E. Laouini, Abderrhmane Bouafia, Souhaila Meneceur, Mohammed L. Tedjani, Sohad A. Alshareef, Hanadi A. Almukhlifi, Khansaa Al-Essa, Ethar M. Al-Essa, Mohammed M. Rahman, Osama Madkhali, Farid Menaa

**Affiliations:** ^1^ Department of Process Engineering, Faculty of Technology, University of El Oued, El-Oued, Algeria; ^2^ Laboratory of Biotechnology Biomaterial and Condensed Matter, Faculty of Technology, University of El Oued, El-Oued, Algeria; ^3^ Department of Chemistry, Faculty of Science, University of Tabuk, Tabuk, Saudi Arabia; ^4^ Department of Chemistry, Jerash University, Jerash, Jordan; ^5^ Department of Civil Engineering, Isra University, Amman, Jordan; ^6^ Center of Excellence for Advanced Materials Research (CEAMR) and Chemistry Department, Faculty of Science, King Abdulaziz University, Jeddah, Saudi Arabia; ^7^ Department of Physics, College of Science, Jazan University, Jazan, Saudi Arabia; ^8^ Department of Biomedical and Environmental Engineering (BEE), Fluorotronics, Inc.-California Innovations Corporation, San Diego, CA, United States

**Keywords:** MgO@AgO/Ag2O NPs, green synthesis, *Portulaca oleracea*, photocatalysis, methylene blue, toluidine blue

## Abstract

**Introduction:** In this paper, MgO@AgO/Ag_2_O nanoparticles were greenly synthesized, the current idea is to replace the harmful chemical technique with an ecofriendly synthesis of metal oxide nanoparticles (NPs) utilizing biogenic sources.

**Methods:** The current investigation was conducted to create silver oxide NPs decorated by MgO NPs (namely, MgO@AgO/Ag_2_O nanocom-posite) using the leaves extract of Purslane (*Portulaca Oleracea*) as the reducing and capping agent. The nanopowder was investigated by means of X-ray diffraction, scanning electron mi-croscope, BET surface area, Fourier transform infrared, and UV-vis spectrophotom-eter studies. XRD studies reveal the monophasic nature of these highly crystalline silver nano-particles. SEM studies the shape and morphology of the synthesis AgO/Ag_2_O and MgO@AgO/Ag_2_O NPs. The presence of magnesium and oxygen was further confirmed by EDS profile.

**Results and discussion:** The surface area was found to be 9.1787 m^2^/g and 7.7166 m^2^/g, respectively. FTIR analysis showed the presence of specific functional groups. UV-vis spectrophotometer studies show the absorption band at 450 nm due to surface plasmon resonance. The results have also indicated the high performance of the greenly synthesized AgO/Ag_2_O NPs and MgO@AgO/Ag_2_O NPs for photocatalytic activity dye degradation (methylene blue and toluidine blue).

## 1 Introduction

With widespread applications in the domains of biology, health, energy, and material science, among others, nanotechnology has emerged as a promising interdisciplinary field of the twenty-first century. Nanomaterials are produced often by a variety of physical and chemical processes that call for high temperatures, vacuum conditions, specialized equipment, and chemical additives (Cai et al., 2022). Due to the use of harmful compounds that remain attached to the synthesized nanoparticles, current improvements in chemical processes for the production of nanomaterials have raised biological dangers to the environment (Yu et al., 2022). Consequently, scientists are presently concentrating on the production of nanomaterials utilizing biogenic sources such as bacteria, algae, and plants. A new age for secure nanobiotechnology has begun because of recent advancements in the green synthesis of nanomaterials, which are rapid, inexpensive, and ecofriendly. Due to their unique physical and chemical features in the domains of biosensors, diagnostic tools, catalysts, anticancer, and antimicrobial agents, metal oxide nanoparticles have recently attracted a lot of attention (Zhang et al., 2020; Ahmad et al., 2021; Wijesinghe et al., 2021a; Wijesinghe et al., 2021b; Gherbi et al., 2022).

In part because of their non-biodegradable and persistent character, the considerable water pollution caused by inorganic (such as heavy metal ions) and organic compounds—such as synthetic organic colors, persistent organic pollutants, antibiotics, etc.—often present significant obstacles. Additionally, these emerging pollutants may have several detrimental effects on human bodies and aquatic microbes (Sharma and Bhattacharya, 2017; Al-Essa and Al-Essa, 2021). The world’s use of synthetic dyes now exceeds several million tons, with numerous industrial sectors including the printing and textile industries contributing to this (Mashkoor and Nasar, 2020). Therefore, solutions for wastewater pretreatment are required to reduce these contamination issues. Scientifically, the adsorption process is the most effective and practical method for purifying water is adsorption (Al-Essa, 2018; Bulgariu et al., 2019). Adsorbents should be created from free, sustainable, and locally accessible sources to achieve competitive efficacy (typically, plant resources) (Bulgariu et al., 2019).

Due to their high conductivity, silver oxide nanoparticles (AgONPs) stand out among the competition as a desirable material for metallic-based conductive filler (Stewart et al., 2017), capacity to encourage cell proliferation and osteogenic differentiation facilitate bone repair (Zhang et al., 2015; Xu et al., 2020), in addition to their capacity to prevent the development of microbial biofilm (Hileuskaya et al., 2020) and maybe defeat microorganisms that are resistant to many drugs (Rai et al., 2012). AgONPs were highly sought-after for application in biosensors due to these special characteristics (Zeng et al., 2014; Al Essa et al., 2021), prostheses (Gallo et al., 2016), and dental materials (Ohashi et al., 2004). The resultant AgONPs, however, are inappropriate for biomedicine use due to the common synthesis technique, which frequently employs toxic chemical reducing agents that are dangerous for human (such as sodium borohydride and hydrazine) (Kolarova et al., 2015; Rónavári et al., 2021). Magnesium oxide nanoparticles (MgONPs) are harmless and reasonably simple to synthesize among the many distinct inorganic metal oxides. MgONPs have received approval from the United States Food and Drug Administration as safe materials (21CFR184.1431) (Martinez-Boubeta et al., 2010). MgONPs, for instance, can reduce indigestion, start the post-activation of bone-repair scaffolds, and function as hyperthermia agents in cancer treatment. Both MgO and AgO NPs can be now produced using “green synthesis” techniques, which offer straightforward, affordable, quick, and non-toxic approaches to address these problems. The use of diverse biological entities, such as plant extract, in green synthesis techniques replaces the use of chemical reducing agents. (Ahmadi et al., 2018; Hamouda et al., 2019; Okaiyeto et al., 2019), and bacteria (Hamouda et al., 2019) produce a high output of somewhat uniform-sized metallic nanoparticles by acting as both reducing and capping agents. Due to their accessibility, safety, and simplicity of synthesis, plant-based extracts have drawn more attention. They don’t require the preservation of microbial cultures (Roy and Das, 2015). The most often reported use so far has been of various plant fruit and leaf extracts. Purslane is a fascinating possibility for this use among many different plants (Bouafia et al., 2020; Bouafia et al., 2021; Bouafia and Laouini, 2021).

The decor is another method for enhancing photocatalytic activity (Wang et al., 2011; Hunge et al., 2021; Yadav et al., 2021). Surface plasmon resonance (SPR), where Ag, Au, and Pt are typically utilized, is favored by decoration with noble metals (Xing et al., 2017), it has many positive benefits since it promotes the absorption of visible light and the activation of energetic charge carriers, many (Sohrabnezhad and Seifi, 2016), achieves a greater separation of charge, this leads to an increase in redox reactions and a higher formation rate of reactive oxygen species (Ong et al., 2013; Kuriakose et al., 2014; Sohrabnezhad and Seifi, 2016). Previous studies have confirmed the photocatalytic activity of AgO and Mg NPs (Balakrishnan et al., 2020). Moreover, prior studies demonstrated that the decoration of AgONPs with other metals NPs results in a significant improvement in the photocatalytic activity comparing to the nondecorated AgONPs (Laouini et al., 2021).

In this study, the degradation of the methylene blue (MB) and toluidine blue (TB) dyes under sunlight was used to examine the photocatalytic capabilities of AgO/Ag_2_O nanoparticles (NPs) coated with MgO. To the best of our knowledge AgO/Ag_2_O and MgO@AgO/Ag_2_O NPs photocatalytic activity was never tested against MB and TB dyes.

## 2 Materials and methods

### 2.1 Reagents

Silver nitrate (AgNO_3_, 98%), Magnesium chloride hydrates (MgCl_2_, 6H_2_O, 98%), MB (C_16_H_18_ClN_3_S, 98%), and TB (C_15_H_16_ClN_3_S, 98%) were purchased from Sigma-Aldrich,Germany. The leaves of purslane were collected from local fields in El Oued, Southeast of Algeria. Distilled water was used in all the experiments.

### 2.2 Preparation of the leaf extract

Fresh and healthy purslane (*Portulaca oleracea*) leaves were collected from nearby farms in the El Oued area (Southeast of Algeria). After properly cleaning them with flowing tap water to get rid of any dirts or other polluted organic materials, they were repeatedly rinsed in de-mineralized water. The fresh leaves were crushed. The leaves components were extracted by mixing 250.0 g of leaves with 900 ml of distilled water in a 1,000 ml glass beaker. The mixture was stirred for 15 min at 70°C. The extract was kept cool and then filtered using the decantation method and stored at 4°C for further use.

### 2.3 Synthesis of AgO/Ag_2_O and MgO@AgO/Ag_2_O NPs

The AgO/Ag_2_O and MgO@AgO/Ag_2_O NPs were synthesized by a green method using plant extract, following modified protocols from previous studies (Abdullah et al., 2020; Bouafia and Laouini, 2020; Bouafia et al., 2021a; Bouafia et al., 2021b; Belaiche et al., 2021; Laid et al., 2021; Laouini et al., 2021; Ben Amor et al., 2022; Daoudi et al., 2022; Djamila et al., 2022; Meneceur et al., 2022; Tedjani et al., 2022). For the synthesis of AgO/Ag_2_O NPs filtered extract was taken and diluted to 1,000 ml with deionized water, 1.7 g of AgNO_3_ was added to 1,000 ml of the extract with continuous stirring, 5 ml diluted hydroxide (0.1 M) was added dropwise to the mixture solution. With controlled and continuous stirring (600 rpm) at 70°C for 4 h, the precursor became brown. The precipitate was then centrifuged and washed several times with deionized water, and dried at 100°C in an oven for 48 h. Finally, the AgO/Ag_2_O NPs were manually grinded into a fine powder.

Nanostructured MgO loaded AgO/Ag_2_O were simply synthesized. Firstly, nanoparticle solution (AgO/Ag_2_O) was prepared by dissolving 0.7 g of (AgO/Ag_2_O) in de-ionized water, the solution maintained in continuous stirring at 70°C for 2 h. Then 70 ml (0.01 M) metal ion (MgCl_2_) solution was added to the nanoparticle solution, mixed thoroughly at 80°C for 2 h, and then dried at 100°C for 48 h.

After drying, the mixture was grinded to obtain a homogeneous powder.

### 2.4 Material characterizations

The employment of the following methods was necessary for the characterization of generated nanoparticles: X-ray diffractometer (XRD), a scanning electron microscope (SEM), EDS, BET surface area analyzer, Fourier transform infrared (FTIR), and UV-vis spectrophotometer.

Using XRD (Rigaka Miniflex 600) and Cu-K radiation with a wavelength of 0.15406 nm in the 2 range 10–80, the crystalline structure of the produced NPs was determined. The morphology of the prepared AgO/Ag_2_O and MgO@AgO/Ag_2_O NPs were verified using SEM (TESCAN VEGA 3) with an accelerating voltage of 10 kV. The BET surface area analyzer (micromeritics ASAP 2020 Plus Version 2.00) was used to capture the N_2_ adsorption isotherm, associated surface area, and pore parameter. A Nicolet iS5 (Thermo Fisher Scientific) was used to perform FTIR measurements on leaves extract, green synthesis AgO/Ag_2_O, and MgO@AgO/Ag_2_O NPs to determine the functional groups operating in the region of 4,000 to 400 cm^−1^. A UV-vis spectrophotometer was used to examine the optical properties of the samples (Shimadzu-1800). The measurement was made at the temperature of 28°C in the 300–900 nm wavelength range. UV-vis spectrometric measurements were performed using a quartz cell and distilled water as a blank solution to determine the stability of AgO/Ag_2_O NPs. The optical gap band energy (Egap) of these materials was calculated using the Tauc Equation considering these findings.

### 2.5 Photocatalytic degradation of MB dye

Based on the samples’ capacity to break down the MB dye in the presence of sunlight, the catalytic activity of the samples was evaluated. The experiment was conducted in a lab using sunlight (the samples were not exposed to direct sunlight). MB stock solution (2.5 × 10^−5^ M) was made. Four samples of 5 ml of dye solution and 5.0 mg of AgO/Ag_2_O NPs were combined and exposed to sunlight in. About 2 ml of the suspensions were taken from the reaction mixture every 15, 30, 60, and 120 min while it was exposed to sunlight, and the suspended particles were then extracted using ultracentrifugation. The same test was conducted using MgO@AgO/Ag_2_O. The absorption obtained at 664 nm in a UV-vis spectrophotometer was used to calculate the rate of dye degradation. Based on the formula, the dye degradation % was evaluated using the Eq. 1:

$$\%\;of\;degradation=\frac{C_{i}-C_{t}}{C_{t}}$$ (1)

where $C_{i}$ and $C_{t}$ (mg/L) are the initial concentration of MB and the concentration of pollutant MB at time t, respectively.

### 2.6 Photocatalytic degradation of TB dye

The photodegradation of TB dye in aqueous solution was investigated under sunlight in the presence of AgO/Ag_2_ONPs and MgO@AgO/Ag_2_O as a photocatalyst, A5.0 mg of the catalyst is added to 50 ml of TB dye solution. This solution is prepared in distilled water at a concentration of (4 × 10^−3^ M). It was then exposed to sunlight at different intervals (10, 20, 30, 40, and 60 min) at a temperature of 28°C and a neutral pH. The catalyst is separated by ultracentrifugation. A spectrophotometer is used for reading the absorbance values at 631 nm.

The equilibrium amount of adsorption is calculated by the Eq. 2:

$$QE=\frac{\left(C_{0}-C_{e}\right)V}{m}$$ (2)

where, QE (mg/g) is the adsorption capacity at equilibrium, $C_{0}$ and $C_{e}$ (mg/L) are the concentration of TB solutions at the start of reaction and the concentration of pollutant TB at time t, respectively. While V stands for the volume of the solution (L), and m is the mass of adsorbent (g).

## 3 Results and discussion

### 3.1 Crystal structure and composition

*Portulaca oleracea’s* medicinal potency is attributed to its complex chemical makeup composition; it is high in primary and secondary metabolites, as well as minerals, vitamins, and other micronutrients.

Purslane is the common name for *Portulaca oleracea L.*, a member of the *Portulaceae* family. A variety of substances have been identified from *Portulaca oleracea*, including flavonoids, alkaloids, polysaccharides, fatty acids, terpenoids, sterols, proteins, vitamins, and minerals. There is a wide range of pharmacological qualities that *Portulaca oleracea* exhibits, including neuroprotective, antibacterial, antidiabetic, antioxidant, anti-inflammatory, antiulcerogenic, and anticancer actions. (Zhou et al., 2015)

To investigate the size and make-up of the crystalline phases, XRD analysis was done.

**FIGURE 1:**
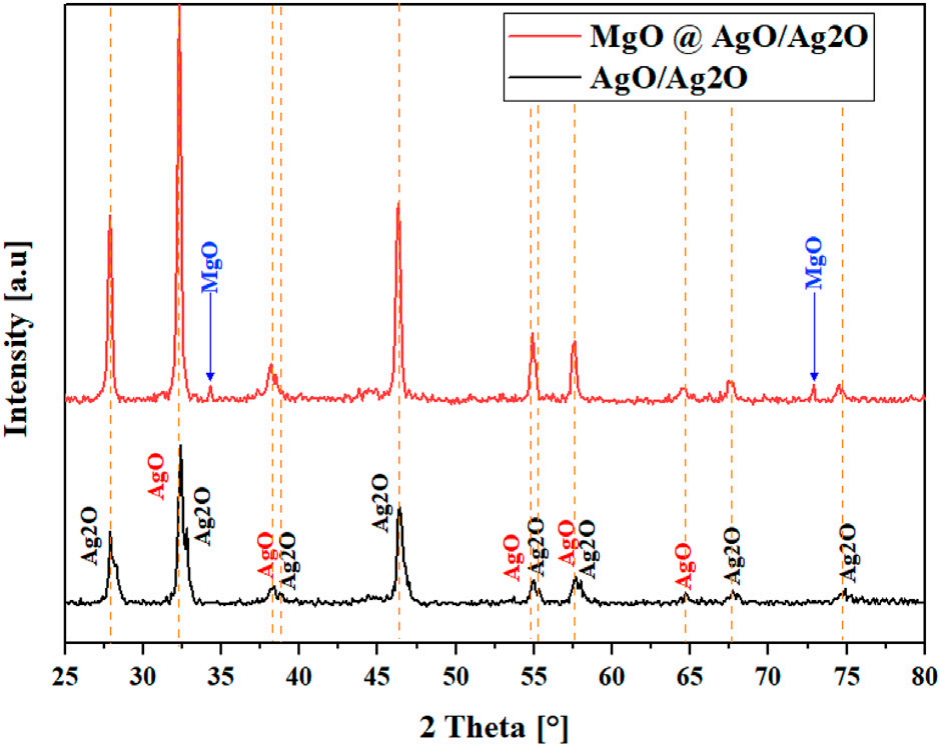
XRD patterns of AgO/Ag_2_O and MgO@AgO/Ag_2_O NPs.

Figure 1 shows the diffraction patterns of AgO/Ag_2_O and MgO@AgO/Ag_2_O. Similar results have already been described in the literature (Deekshitha and Shetty, 2021), regarding the peaks of Ag_2_O. Moreover, It is intriguing to see that there are two distinct peaks that can be attributed to MgO NPs. According to the Debye-Scherrer equation, the crystallite size was determined to be 22.16 nm for AgO/Ag_2_O and 21.60 nm for MgO@AgO/Ag_2_O NPs, respectively. The results are shown below in Table 1.

**TABLE 1:** Quantification and average crystallite size of AgO/Ag_2_O and MgO@AgO/Ag_2_O NPs.

Crystallite size (nm)	Presented Phases			Lattice parameters	Crystal system	COD Entry	References
	Name	Amount (%)	Formula				
21.60	Magnesium Oxide Periclase	31.1	MgO	Space Group: Fd-3m E a = 8.12 Å *α* = 90.0000° b = 8.12 Å *β* = 90.0000° c = 8.12 Å *γ* = 90.0000°	Cubic	96-500-0226	Freund and Farmer, (1974)
	Silver oxide	15.3	AgO	Space Group: P 1 (#1-1) a = 4.21 Å *α* = 90.0000° b = 4.21 Å *β* = 90.0000° c = 4.21 Å *γ* = 90.0000°	Cubic	01-076-1489	Stehlik et al. (1959)
		55.7	Ag_2_O	Space Group: P 1 (#1-1) a = 4.71 Å *α* = 90.0000° b = 4.71 Å *β* = 90.0000 c = 4.71 Å *γ* = 90.0000°	Cubic	00-003-0796	Tulsky and Long, (2001)
22.16	Silver oxide	22%	AgO	Space Group: P 1 (#1-1) a = 4.21 Å *α* = 90.0000° b = 4.21 Å *β* = 90.0000 c = 4.21 Å *γ* = 90.0000°	Cubic	01-076-1489	Stehlik et al. (1959)
		78%	Ag_2_O	Space Group: P 1 (#1-1) a = 4.71 Å *α* = 90.0000 b = 4.71 Å *β* = 90.0000 c = 4.71 Å *γ* = 90.0000°	Cubic	00-003-0796	Tulsky and Long, (2001)

### 3.2 Morphological investigation

**FIGURE 2:**
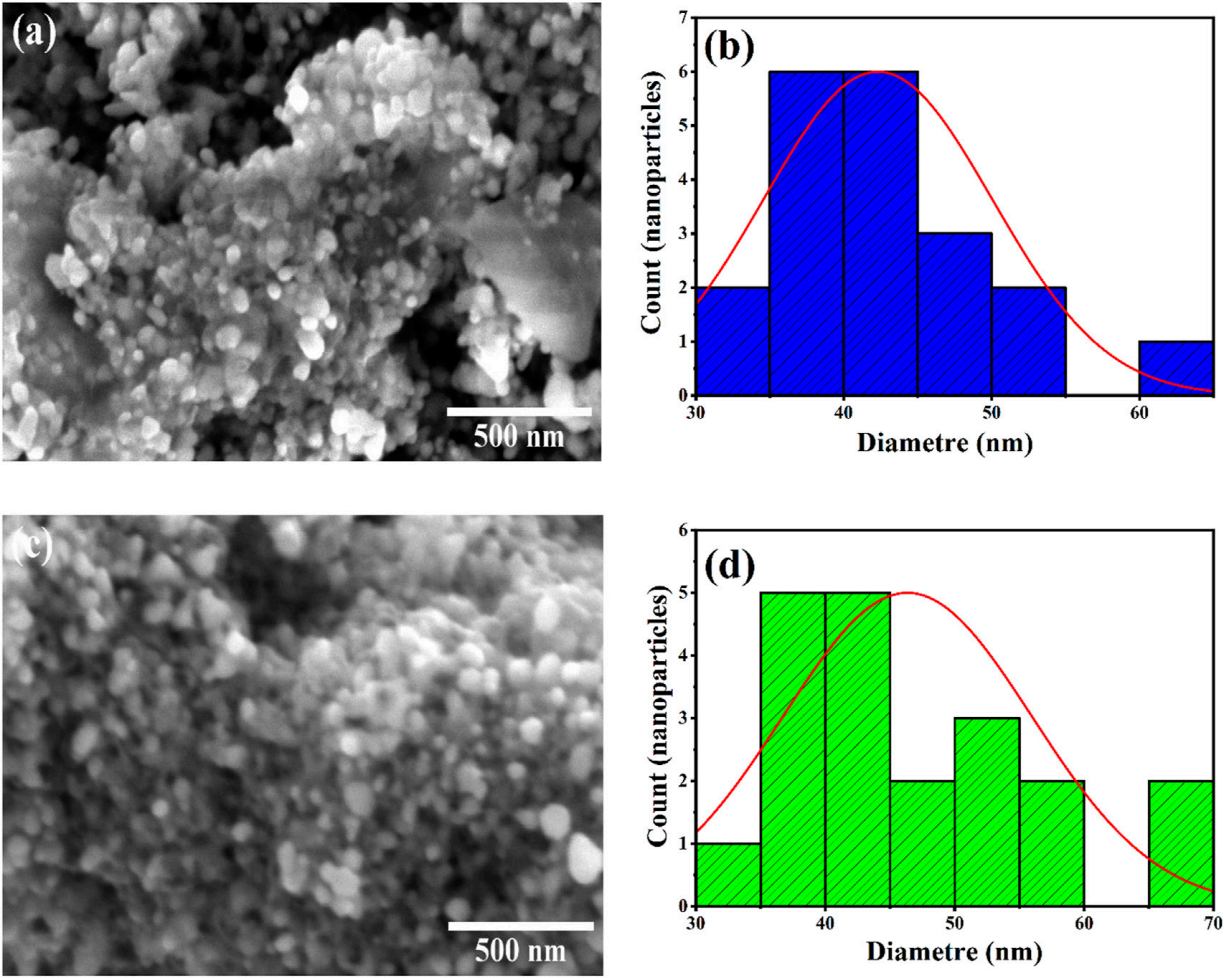
SEM images and particle size distributions: **(A,B)** AgO/Ag_2_O NPs, **(C,D)** MgO@AgO/Ag_2_O NPs.

SEM was used to study the formation of the prepared AgO/Ag_2_O and MgO@AgO/Ag_2_O NPs (Figures 2A,C, respectively), and their morphological size (40–50 nm in average) (Figures 2B, D, respectively). The MgO@AgO/Ag_2_O NPs were oval and spherical. Most of the MgO@AgO/Ag_2_O NPs were placed as aggregated which may explain the slight increase in size of these NPs compared to AgO/Ag_2_O NPs; also, a few individual particles were also observed (Gosens et al., 2010).

**FIGURE 3:**
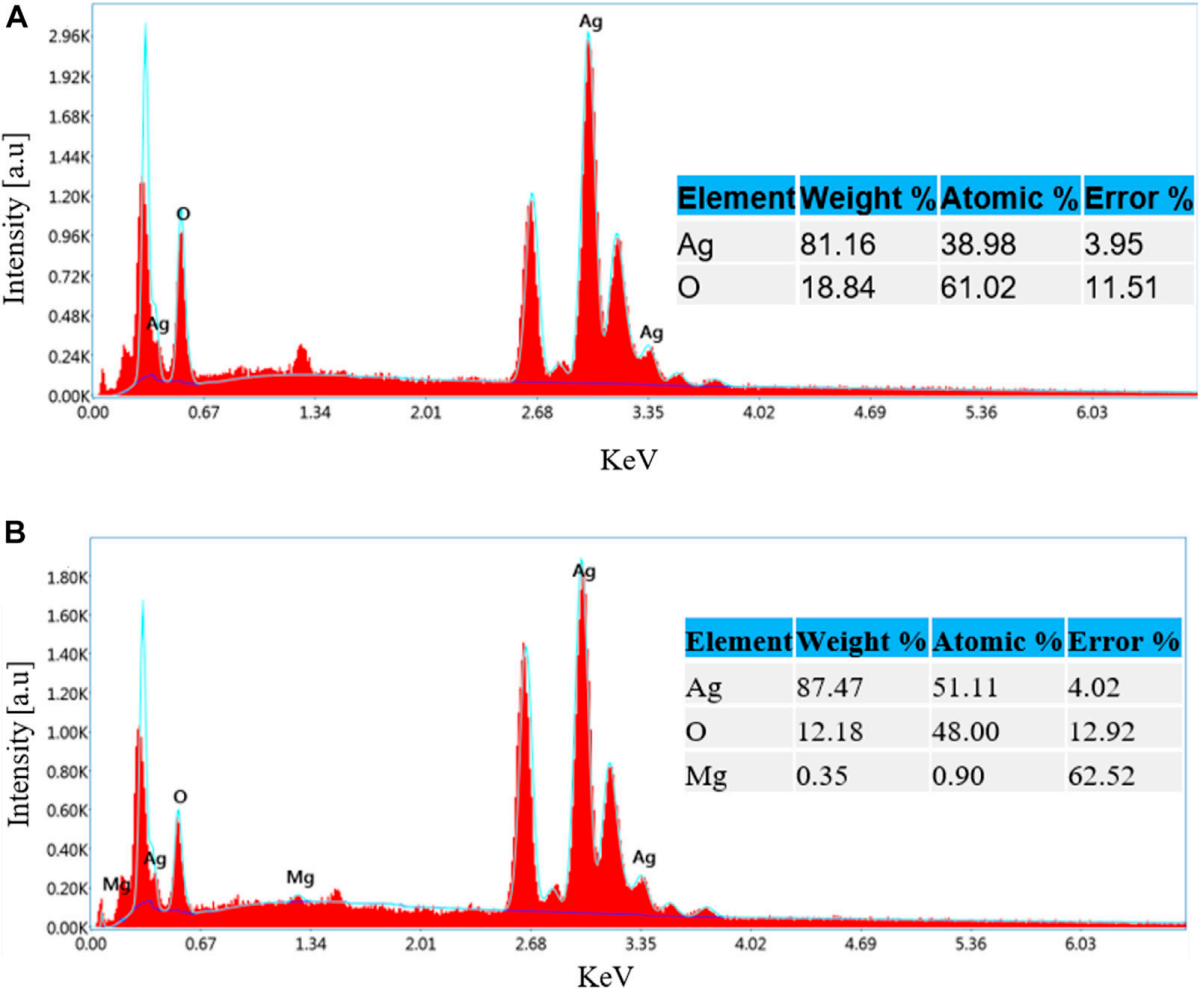
EDS of: **(A)** AgO/Ag_2_O and **(B)** MgO@AgO/Ag_2_O NPs.

Further analysis by EDS of the prepared AgO/Ag_2_O and MgO@AgO/Ag_2_O NPs (Figures 3A,B, respectively). The data associated to MgO@AgO/Ag_2_O NPs, confirm the presence of silver, oxygen, and magnesium, with a weight percentage of approximately 87.47% Ag, 12.18% O and 0.35% Mg. It is concluded that all nanoparticles were mixed together. In this way, the aimed ternary oxides nanoparticle system containing MgO, AgO, and Ag_2_O NPs was obtained.

The MgO@AgO/Ag_2_O NPs sample’s EDAX analysis revealed only clear peaks for the elements Ag, Mg, and O; no additional peaks could be observed, proving that the powder was produced without any impurities.

### 3.3 N_2_ adsorption–desorption isotherm

**FIGURE 4:**
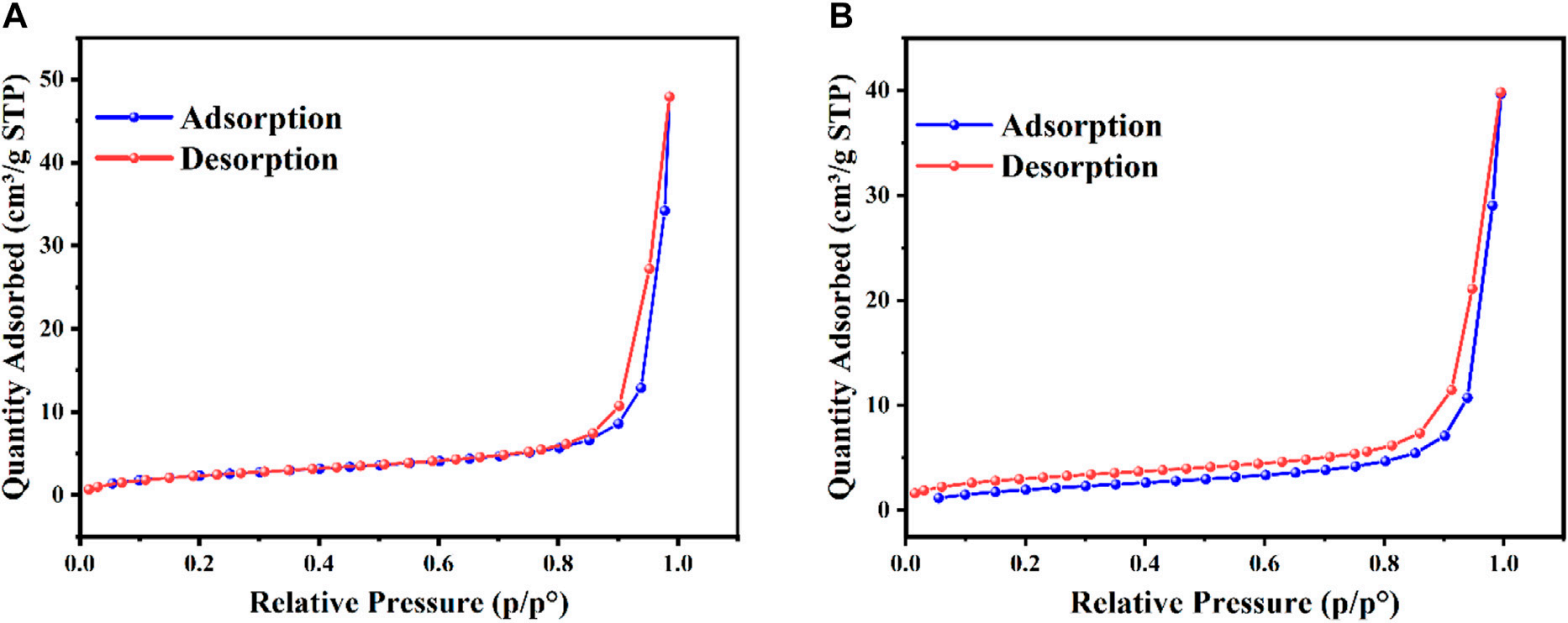
Nitrogen adsorption-desorption isotherms: **(A)** AgO/Ag_2_O, **(B)** MgO@AgO/Ag_2_O NPs.

Figure 4 shows the N_2_ adsorption-desorption isotherm of 1) AgO/Ag_2_O and 2) MgO@AgO/Ag_2_O NPs. The specific BET surface area was found to be 9.1787 m^2^/g and 7.7166 m^2^/g (Table 2). The average pore diameter is 3.1 nm, which demonstrates the existence of mesopores. The pore size between 2.89018 and 2.83265 nm shows that the AgO/Ag_2_O and MgO@AgO/Ag_2_O NPs, belong to mesoporous materials (2–50 nm), this result can be easily verified by SEM images (Figure 4). The noticeable improvement, in the specific surface area value, proves the enormous photocatalytic activity of the MgO@AgO/Ag_2_O NPs.

**TABLE 2:** BET surface area, porosity, and particle size of AgO/Ag_2_O and MgO@AgO/Ag_2_O NPs.

Samples		AgO/Ag_2_O	MgO@AgO/Ag_2_O
Surface Area	BET Surface Area	9.1787 m^2^/g	7.7166 m^2^/g
	Langmuir Surface Area	376.2072 m^2^/g	279.6492 m^2^/g
Pore Volume	Single point adsorption total pore volume of pores less than 40.3122 nm diameter at p/p° = 0.950000000	0.021557 cm³/g	0.020119 cm³/g
	Single point desorption total pore volume of pores less than 40.3122 nm diameter at p/p° = 0.950000000	0.039853 cm³/g	0.033269 cm³/g
Pore Size	BJH Adsorption average pore diameter (4V/A)	3.10194 nm	3.15833 nm
	BJH Desorption average pore diameter (4V/A)	2.89018 nm	2.83265 nm
Nanoparticle Size	Average Particle Size	65.3689 nm	77.7541 nm

The figure below presented (Figure 4) depicts the nitrogen adsorption isotherms on the surface of the AgO/Ag_2_O and MgO@AgO/Ag_2_O NPs. The figures clearly show that:

- All compounds of these nanoparticles belong to mesoporous materials.
- The compound MgO@AgO/Ag_2_O NPs presents the best catalytic behavior with concerning the adsorption of N_2_.

### 3.4 FTIR study

**FIGURE 5:**
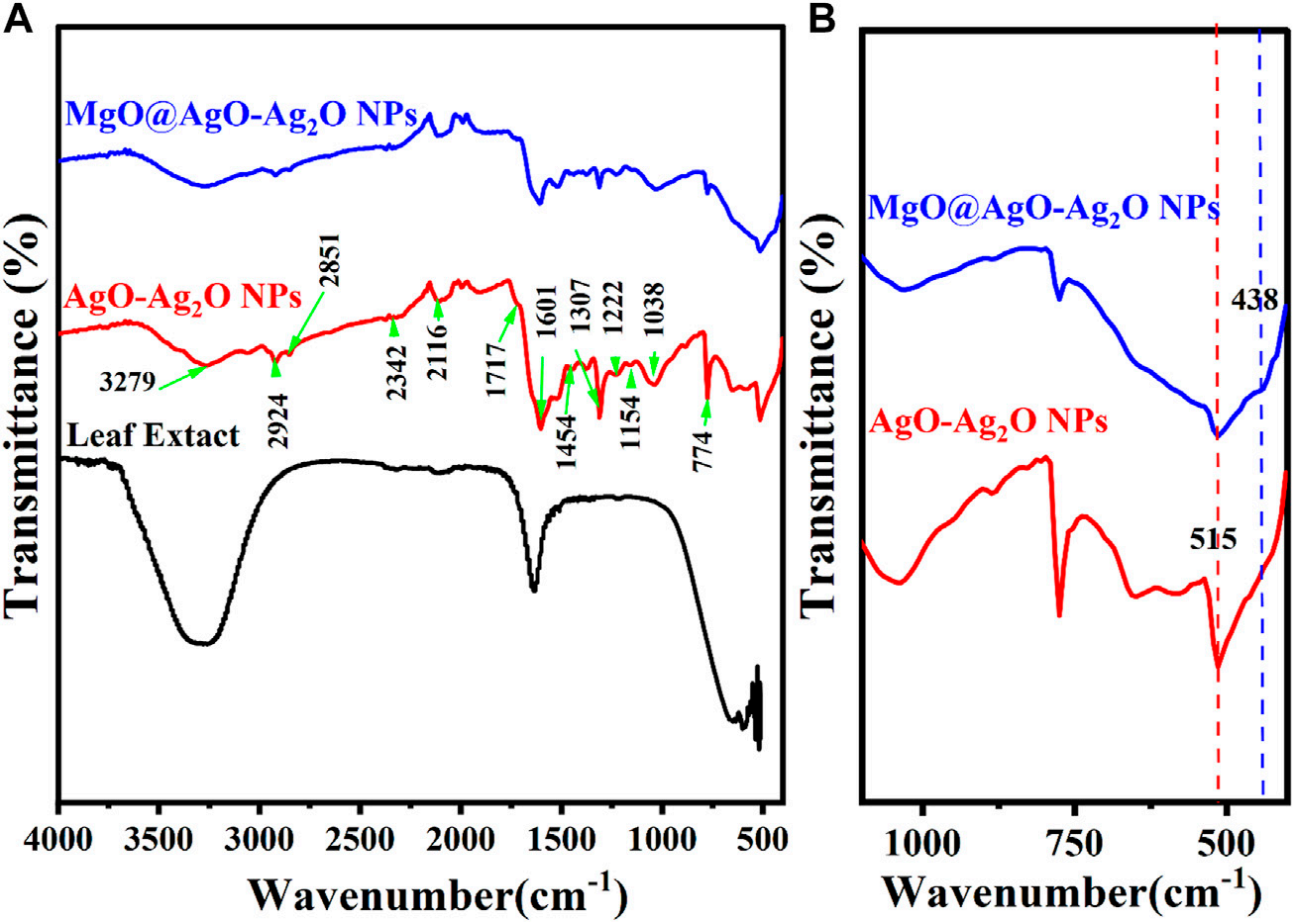
**(A)** FTIR spectra of Purslane (Portulaca Oleracea) leaf extract, AgO/Ag_2_O, and MgO@AgO/Ag_2_O; **(B)** zoomed view range (400 to 1,100 cm^−1^).

As a result of the aliphatic C-H stretching vibration of hydrocarbon chains and N-H bending vibration, the spectral peaks at 2,924, 2,851, and 1,454 cm^1-^ (Ananthi et al., 2018; Hamouda et al., 2019) are shown in Figure 5.

Peaks centered at 1,340, 1,224, 1,142, and 1,024, and cm^−1^ in the range 1,300–1,000 cm^−1^ are caused by stretching vibrations of C-O groups in anhydrides, esters, ethers, alcohols, and phenols, C-O-H groups in alcohols and phenols, and C-N groups in amines. The vibration of the C=C group of alkenes, the N-H groups of amides, & the amine salts is represented because of the stretch that was seen at 1,603 cm^−1^. It is possible that the broad stretch in the 3,400–2,400 cm^−1^ range, which is centered at 3,283 cm^−1^, is caused by the stretching vibrations of amines, amine salts, sulfonamides, alkenes and alkanes, amines’ N-H and C-H, and carboxylic acids’ C=O. Nitriles, aromatic rings, and aldehydes may all be appeared as other weak bands on the graph Thus, the results of the FTIR investigation show that the main contributing factors to the reduction of Ag^+^ ions to Ag^0^ nanoparticles in the floral extract were the -C=O (carboxyl), -OH (hydroxyl), and N-H (amine) groups (Chekuri et al., 2015). The interaction of these functional groups with the AgONPs may be responsible for the shifting of these peaks (Hamouda et al., 2019).

The FTIR spectra show that the Mg-O stretching frequency of MgO corresponds to the IR peak at 436 cm^−1^ (Sahoo et al., 2020).

Additionally, the disappearance of the phenolic compound-associated absorbance bands 3,278, 2,924, 2,851, and 1,454 cm^−1^ after the synthesis of MgO@AgO/Ag_2_O NPs leads us to conclude that the *Portulaca oleracea L*. leaf extract contains phytochemicals like alcohols, aldehydes, alkanes, epoxy groups, and ether groups that may be responsible for the nucleation process to reduce precursor from M^+^ to M^0^.

### 3.5 Optical properties

**FIGURE 6:**
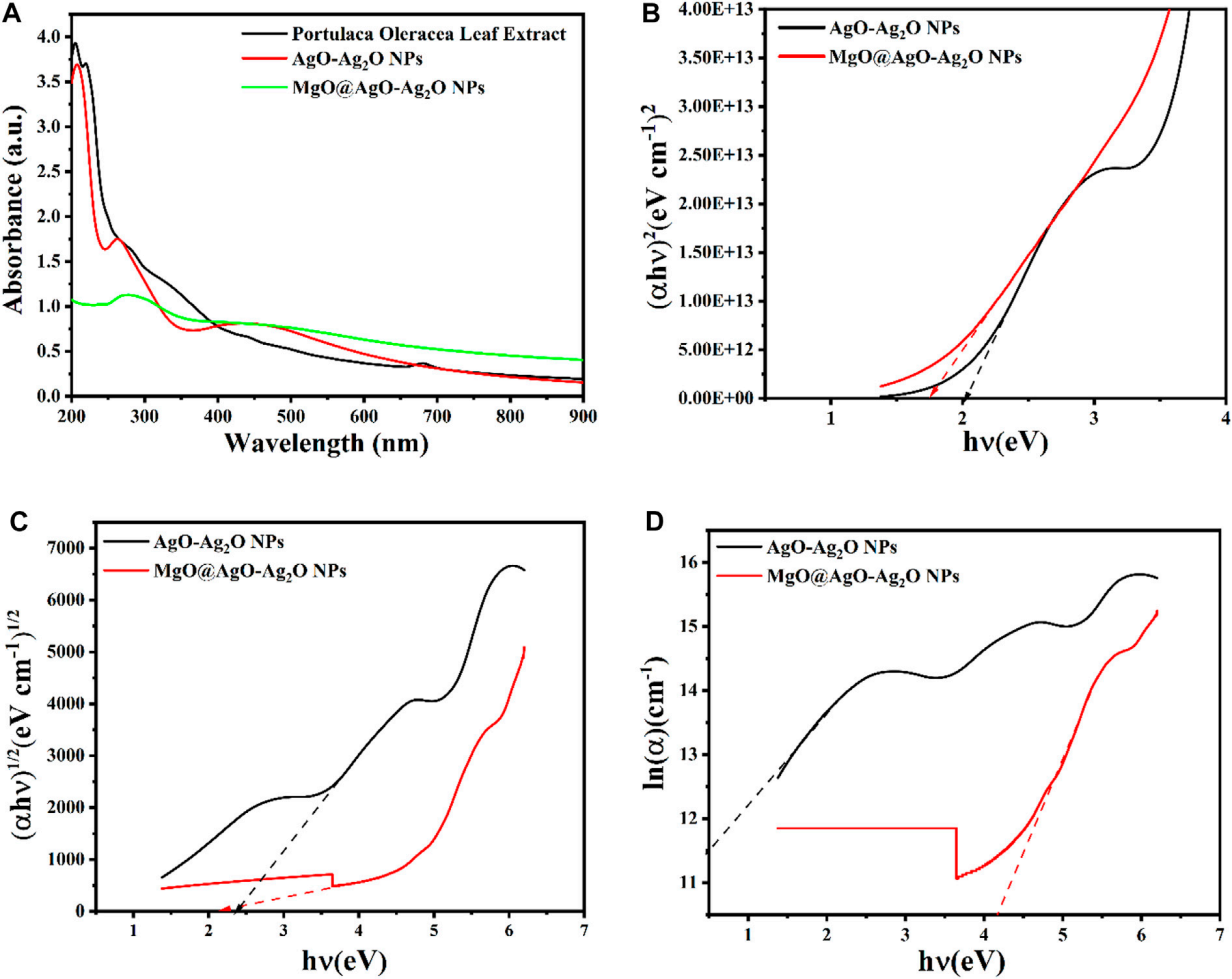
UV-vis spectra of **(A)** Extract, AgO/Ag_2_O, and MgO@AgO/Ag_2_O **(B)** Optical energy gap for direct, **(C)** Indirect transitions, and **(D)** Urbach energy.

The formation of AgONPs was achieved within 90 min after adding 1.7 g of AgNO_3_ to the stirred and heated extract. The process did not require the addition of external additives. In addition, bioactive molecules in leaf extracts interact with silver ions (Ag+) and fuse neighboring small particles into nanoparticles, leading to nucleation of (Ag◦) atoms and promoting the nanoparticle growth (Sangar et al., 2019). The formation of AgONPs was confirmed visually by the color change of the reaction mixture from pale green to dark brown. UV-vis spectral scans are commonly implemented to confirm the formation of metal nanoparticles in aqueprobleous solution. Similarly, the product obtained exhibited a surface plasmon resonance (SPR) peak at 240 nm, as a characteristic of AgONPs (Aisida et al., 2019) (Figure 6A). Purslane extract exhibited a peak at approximately 240 nm attributed to the properties of bioactive reducing agent molecules.

To further improve the properties, we synthesized hybrid nanostructures MgO@AgO/Ag_2_O containing 10 mM MgCl_2_. Magnesium incorporation into the AgO/Ag_2_O NPs solution was confirmed by UV-vis spectral analysis. However, the reduced SPR peak intensity is related to confirmation of MgO capping on the AgNP surface (Sathiyaseelan et al., 2020).

The optical band gaps of the biosynthesized NPs were estimated using the absorption spectra of both direct and indirect transitions. The Tauc’s equation (Eq. 3) was used to obtain the optical bandgap (Eg) of AgO/Ag_2_O and MgO@AgO/Ag_2_O NPs (Soltan et al., 2017; Lassoued et al., 2018).

$$\left(\alpha hv\right)=A{\left(hv-E_{g}^{opt}\right)}^{n}$$ (3)

According to Lambert–Beer–Bouguer Law, the absorption coefficient (Eq. 4) is given as (Kilic et al., 2019):

$$\alpha =\frac{A}{t}\;2.303$$ (4)

here, a represents the absorbance and t is the of quartz cuvette width (10 mm).

For the direct transition Tauc plots were plotted using ${\left(\alpha h\nu \right)}^{2}$ against hν, meanwhile ${\left(\alpha h\nu \right)}^{1∕2}$ against hν were used to plot the indirect transition graph for each sample. Were hν, h , ν and *α* represents the photon energy, Planck’s constant, the photon frequency, and the absorption coefficient respectively.

The indirect optical bandgap values of the biosynthesized AgO/Ag_2_O and MgO@AgO/Ag_2_O NPs varied from 2.35 to 2.17 eV (Figure 6C), while the direct bandgap values varied from 2.02 to 1.75 eV (Figure 6B). The direct optical bandgap of the synthesized sample increased with decoration, while the indirect optical bandgap decreased. Higher bandgap values may indicate the presence of confinement effects in the manufactured product. Such high bandgap values of prepared nanostructures make them suitable for electro-optical devices (Shakir et al., 2009; Shkir et al., 2018).

The Urbach energies (Eq. 5) can be estimated exploiting the slope of the absorption edge in the semi-logarithmic plot (Figure 6D).

$$\ln\alpha =\frac{h\nu }{E_{u}}+constant\;\left(\ln\alpha_{0}\right)$$ (5)

here, $E_{u}$ signifies the Urbach energy.

During our investigation, we observed changes in the Urbach energy of the biosynthesized NPs. These changes occurred due to the influence of MgO. Where an increase in the Urbach energy (from 0.379 to 0.666 eV) was observed upon MgO incorporation, indicating a distortion of the structural order. The Urbach energy characterizes the homogeneity and stability of NPs. The lower the Urbach energy, the more uniform and stable the nanoparticles, and *vice versa*. The increasing AgO addition to the glass structure explains that the structure has become disordered and unstable. This result is consistent with the literature (Table 3) (Işsever et al., 2019).

**TABLE 3:** Direct, indirect optical band gaps, and Urbach energies of synthesized NPs.

Samples	Urbach energy (eV)	Direct optical bandgap (eV)	Indirect optical bandgap (eV)
AgO/Ag_2_O	0.379	2.02	2.35
MgO@AgO/Ag_2_O	0.666	1.75	2.17

### 3.6 Photocatalytic activity of AgO/Ag_2_O and MgO@AgO/Ag_2_O for azo dye degradation

The organic MB dye was used to measure the catalytic activity of AgO/Ag_2_O and MgO@AgO/Ag_2_O NPS by measuring the decomposition rate of MB. Selected at 664 nm.

**FIGURE 7:**
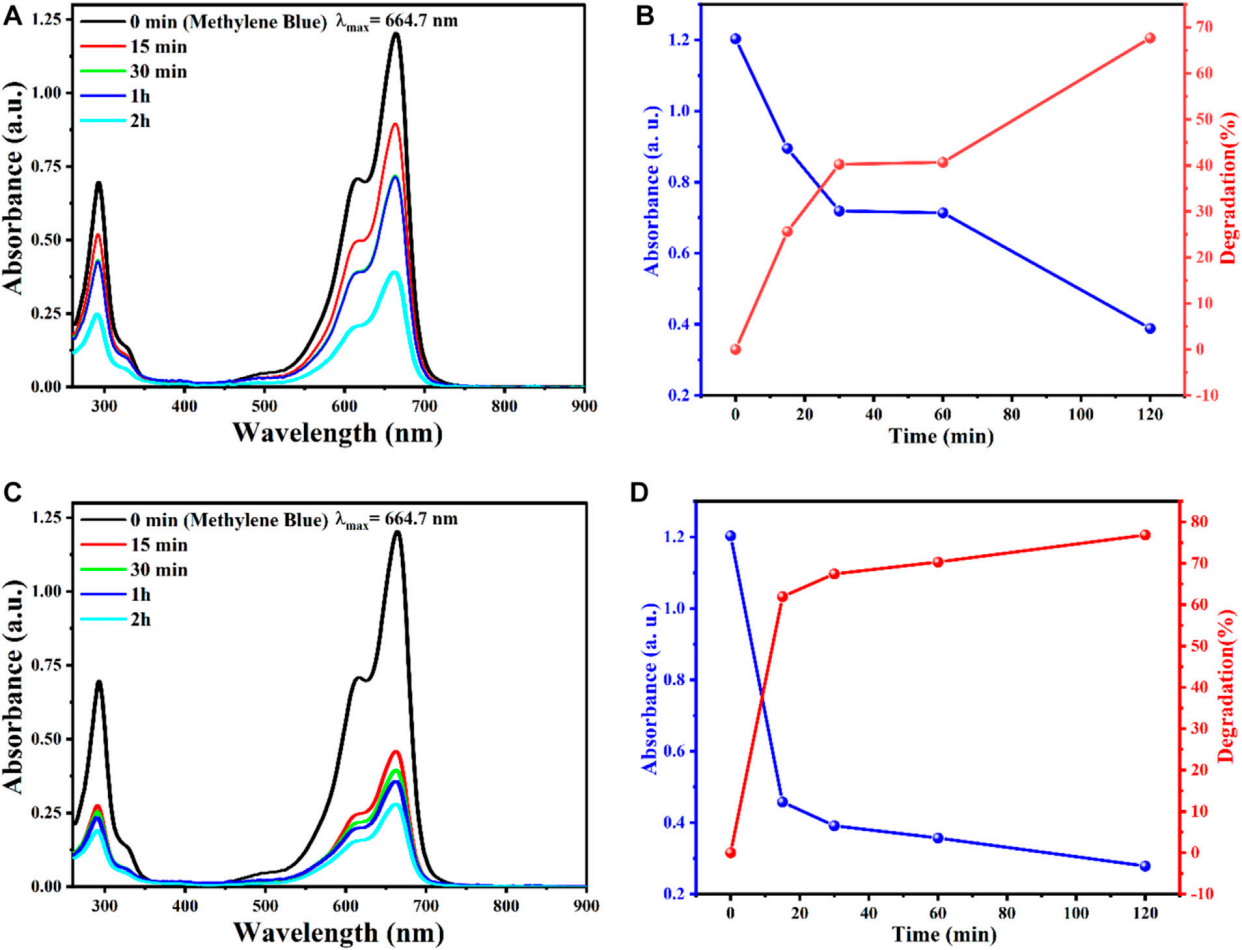
The effect reaction time on the degradation of MB, obtained by **(A)** AgO/Ag_2_O, Absorbance and degradation versus time: **(B)** AgO/Ag_2_O, **(C)** MgO@AgO/Ag_2_O; **(D)** MgO@AgO/Ag_2_O NPs catalyzed degradation of MB dye.

The percentage of MB degradation by AgO/Ag_2_O and MgO@AgO/Ag_2_O NPs in the presence of sunlight is shown in (Figures 7B, D). The results showed that the percentage MB decomposition values of synthesized AgO/Ag_2_O and MgO@AgO/Ag_2_O NPs changed from 67.71363% to 76.86329% in the presence of sunlight.

According to (Figures 7A, C), magnesium decoration had a significant effect on the photocatalytic efficiency of AgO/Ag_2_O powders. The photocatalytic efficiency of AgO/Ag_2_O powder was increased by magnesium decoration. As it can been seen, the photocatalytic efficiency was directly proportional to the exposure duration (time).

This result is caused by surface plasmon resonance (SPR), resulting in increased redox reactions and a higher rate of reactive oxygen species generation. Additionally, heterojunctions structured between AgO/Ag_2_O and MgO@AgO/Ag_2_O NPs act to increase charge carrier separation, thereby providing highly reactive species that function in photocatalysis (Wang et al., 2011; Ong et al., 2013; Paula et al., 2019).

**FIGURE 8:**
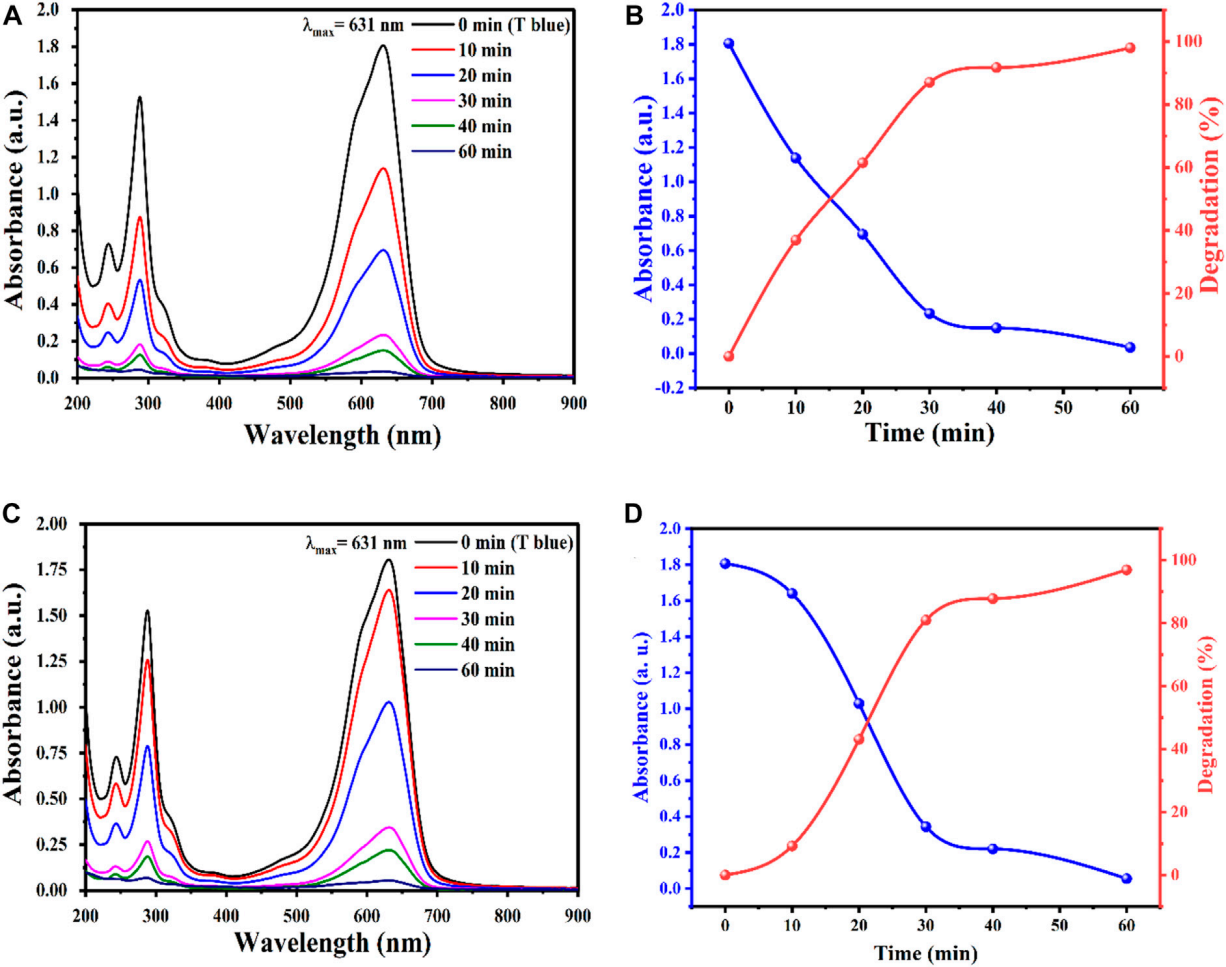
The effect of reaction time on degradation of TB, obtained by **(A)** AgO/Ag_2_O, Absorbance and degradation versus time: **(B)** AgO/Ag_2_O, **(C)** MgO@AgO/Ag_2_O; **(D)** MgO@AgO/Ag_2_O NPs catalyzed degradation of TB dye.

**FIGURE 9:**
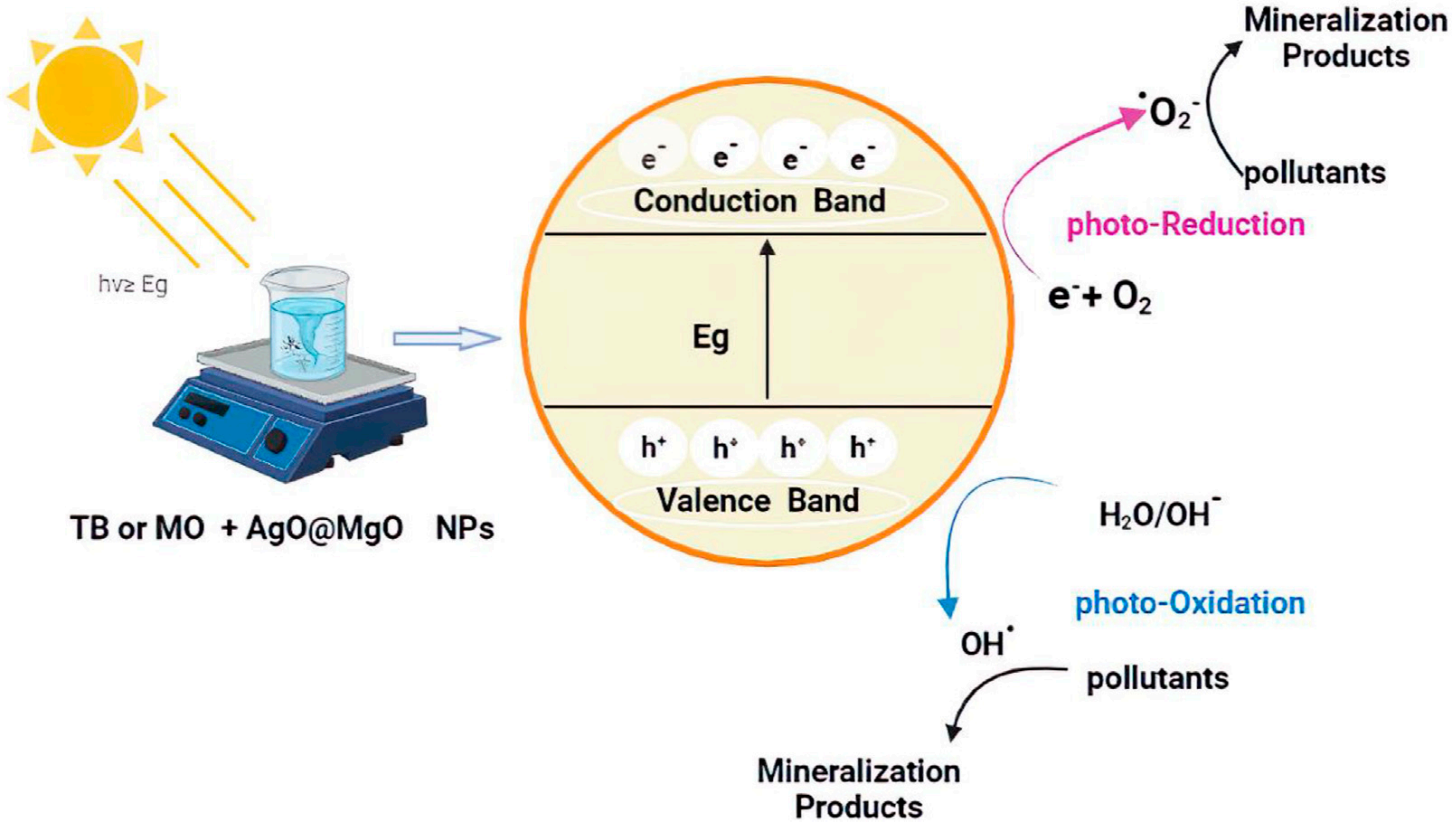
MB and TB photocatalytic mineralization mechanism on the surface of AgO/Ag_2_O and MgO@AgO/Ag_2_O.

The effect of photocatalyst decomposition is shown in the Figure 8. Irradiation of NPs affects the degradation of pollutant TB. This effect may be due to the availability of sufficient energy to excite electrons from the valence band to the conduction band. The band gap energy values for AgO/Ag_2_O and MgO@AgO/Ag_2_O NPs (2.02 eV to 1.75 eV) justify the reduction of the band gap energy. The reaction mechanism is shown in Figure 9.

Wastewater purification remains a major environmental issue due to the increasing and widespread spread of non-degradable pollutants in water, Therefore, this study presents the optimization of photocatalytic degradation of TB dye in an aqueous medium by photocatalyst, under sun irradiation. The obtained results revealed that most of the TB were removed within 60 min at a rate of 98%.

Photolysis is a chemical reaction in which a chemical substance is broken down by photons, whereas photocatalysis is defined as the acceleration of a photoreaction in the presence of a catalyst. Surface chemistry peculiar to this family of chemicals influences how electron donors and acceptors interact with metal oxide semiconductors. Light is absorbed by an absorption substrate during catalysed photolysis. For electrons to be promoted from the valence band (VB) to the conduction band (CB), forming electron-hole (e^−^/h^+^) pairs, photocatalyst on semiconducting oxides must absorb photons with energy equal to or greater than the oxide’s band gap (Yao et al., 2008; Mittal et al., 2009), as shown in the Eq. 6.

The following steps sum up the mechanism of oxidation for the photodegradation process of the pollutant MB and TB:

Step 1: The radical anion is formed Eq. 6:

$$e_{\left(CB\right)}^{-}+O_{2}\overset{AgO/Ag2O\;or\;MgO@AgO/Ag2O}{\to }\;O_{2}^{-\cdot }$$ (6)

Step 2: The OH radical group appears by the hole (7–11);

$$h_{\left(VB\right)}^{+}+H_{2}O\overset{AgO/Ag2O\;or\;MgO@AgO/Ag2O}{\to }\;H^{+}+{OH}^{-}$$ (7)

$$H^{+}+{\dot{O}}_{2}^{-}\overset{\;}{\to }{H\dot{O}}_{2}$$ (8)

$$H{\dot{O}}_{2}+H{\dot{O}}_{2}\;\overset{\;}{\to }\;H_{2}O_{2}+O_{2}$$ (9)

$$H_{2}O_{2}\overset{\;\mathrm{hv}}{\to }2O\dot{H}$$ (10)

Step 3: The organic contaminants are oxidized either through direct contact with the holes or by the main radicals and OH^**·**^ (Eq. 11);

$$Toluidine\;blue\;or\;Methylene\;blue+OH\overset{h^{+}}{\to }Mineralisation\;products$$ (11)

## 4 Conclusion

The catalytic activity of AgO/Ag_2_O and MgO@AgO/Ag_2_O NPs was evaluated based on the rate of degradation of organic dye pollutant. Experimental data illustrated that the percentage of MB degradation values of the synthesized AgO/Ag_2_O and MgO@AgO/Ag_2_O NPs changed from 67.71 to 76.86%, and 98% of TB degradation. Furthermore, under environmental circumstances, the synthesized AgO/Ag_2_O and MgO@AgO/Ag_2_O NPs display strong photocatalytic activity for dye degradation of MB and TB stains. AgO/Ag_2_O and MgO@AgO/Ag_2_O NPs have been found to be useful in the treatment of wastewater (dye degradation). It has proven its ability to purify water from all suspended impurities and remove chemical dyes, and there is a possibility to apply photocatalytic methods to purify water contaminated with hydrocarbons.

## Data Availability

The original contributions presented in the study are included in the article/Supplementary Material, further inquiries can be directed to the corresponding authors.

## Abbreviations

NPs, Nanoparticles; MB, Methylene blue; TB, toluidine blue.
